# Combining 1,4-dihydroxy quininib with Bevacizumab/FOLFOX alters angiogenic and inflammatory secretions in ex vivo colorectal tumors

**DOI:** 10.1186/s12885-020-07430-y

**Published:** 2020-10-02

**Authors:** Susan A. Kennedy, Maria E. Morrissey, Margaret R. Dunne, Fiona O’Connell, Clare T. Butler, Mary-Clare Cathcart, Amy M. Buckley, Brian J. Mehigan, John O. Larkin, Paul McCormick, Breandán N. Kennedy, Jacintha O’Sullivan

**Affiliations:** 1 Department of Surgery, Trinity Translational Medicine Institute, St. James’s Hospital, Trinity College Dublin, Dublin 8, Ireland; 2 UCD Conway Institute & UCD School of Biomolecular and Biomedical Science, University College Dublin, Dublin 4, Ireland; 3 GEMS, St. James’s Hospital, Dublin 8, Ireland

**Keywords:** Angiogenesis, Colorectal cancer, Cysteinyl leukotriene, Angiopoietin-TIE-2 signaling, Combination therapy

## Abstract

**Background:**

Colorectal cancer (CRC) is the second most common cause of cancer-related mortality worldwide with one in every five patients diagnosed with metastatic CRC (mCRC). In mCRC cases, the 5-year survival rate remains at approximately 14%, reflecting the lack of effectiveness of currently available treatments such as the anti-VEGF targeting antibody Bevacizumab combined with the chemotherapy folinic acid, fluorouracil and oxaliplatin (FOLFOX). Approximately 60% of patients do not respond to this combined treatment. Furthermore, Bevacizumab inhibits dendritic cell (DC) maturation in poor responders, a key process for tumor eradication.

**Method:**

Following drug treatment, secreted expression levels of angiogenic and inflammatory markers in tumor conditioned media generated from human ex vivo colorectal tumors were measured by ELISA. Dendritic cell phenotypic and maturation markers were assessed by flow cytometry.

**Results:**

Our novel compound, 1,4-dihydroxy quininib, acts in an alternative pathway compared to the approved therapy Bevacizumab. 1,4-dihydroxy quininib alone, and in combination with Bevacizumab or FOLFOX significantly reduced TIE-2 expression which is involved in the promotion of tumor vascularization. Combination treatment with 1,4-dihydroxy quininib significantly increased the expression level of DC phenotypic and maturation markers.

**Conclusion:**

Our results indicate the anti-angiogenic small molecule 1,4-dihydroxy quininib could be an alternative novel treatment in combination therapy for CRC patients.

## Background

As colorectal cancer (CRC) is the third most common cancer, and the second most common cause of cancer-related mortality worldwide, it is a leading public health concern [1–3]. In 2018 alone, more than 1.8 million people were diagnosed with CRC and it attributed to approximately 862,000 deaths worldwide [3]. The overarching problem in this patient population is at the time of diagnosis, one in five patients will be diagnosed with metastatic CRC (mCRC). In the last decade in the US, the estimated 5-year survival for mCRC has increased by a mere 4%, from 10% in 2008 [4] to 14% in 2019 [5]. While an evaluation of the incidence rates of CRC in the USA shows a decreasing trend between 2000 and 2014 [1] what is most worrying, is that in the last 60 years, CRC has been on the rise in the under 50 population [1]. In some countries the incidence of young CRC is as high as 35–50% [6]. In particular, in the USA in males under 50, CRC is the second leading cause of cancer deaths, while in women under 50, it is the fourth leading cause of cancer deaths [7]**.**

In order for a tumor to survive and thrive within its environment it relies on the process of angiogenesis. The increased demand for oxygen and nutrients from a developing tumor (the angiogenic switch) causes the release of proangiogenic factors such as vascular endothelial growth factor (VEGF), fibroblast growth factor (FGF) and angiopoietins from the tumor microenvironment to promote tumor vascularization [8–10]. During angiogenesis, perivascular cells are required for blood vessel formation, a process in which the angiopoietin-TIE signalling pathway plays an important role [8, 11]. Angiogenesis and inflammation have been described as being mutually dependent processes [12]. It is however, the orchestrated communication and signalling of immune and inflammatory cells that contributes to angiogenesis and metastasis [12]. The initiation and progression of cancer relies on the participation of inflammatory mediators such as TGF-β, TNF-α, IL-10 and IL-6 [13]. Dendritic cells (DC) play a prominent role in this process through their ability to regulate inflammatory responses via their orchestration of adaptive immunity, and release of chemokines and cytokines [14–16].

Targeting angiogenesis therapeutically diminishes a growing tumor of essential nutrients and oxygen it requires to fulfil its metastatic potential. Multiple Food and Drug Administration (FDA) approved therapeutic interventions exist for the treatment of CRC, many of which aim to inhibit tumor angiogenesis by targeting the VEGF pathway, e.g. Nintedanib (Boehringer Ingelheim), Cyramza (Eli Lilly), Sunitinib (Pfizer), Regorafenib (Bayer), and Bevacizumab (Roche) the overall impact of these therapies remains extremely poor. In some cases, the median overall survival and progression free survival remains at less than 6 months. Despite the fact the VEGF targeting monoclonal antibody Bevacizumab has been approved by the FDA since 2004 for use in combination with fluorouracil (5-FU) in patients with mCRC [4], a meta-analysis of ten-studies demonstrated that treatment with Bevacizumab can cause an increase in gastrointestinal perforation 3-fold higher compared to control, and can also cause increased hypertension, thrombosis and bleeding [17]. More importantly, some patients do not respond to Bevacizumab for reasons that are not clearly understood [18]. Drugs targeting the angiopoietin-TIE pathway are also in clinical development and viewed as a potential complementary therapy to VEGF targeting treatments [19]. Additional approved drugs are also available in the mCRC area in particular those targeting epidermal growth factor receptor (EGFR) inhibition. Both Cetuximab and Panitumumab have been approved but only show clinical benefit in 10–20% of patients who present with wildtype KRAS status [20]. All of which demonstrates the urgent need to develop novel anti-angiogenics which also beneficially impact the immune environment.

Previous work from our group identified quininib from a chemical screening library as a lead compound that possessed anti-angiogenic properties in vitro*,* in vivo and ex vivo [21]*.* Further studies of quininib analogues revealed 1,4-dihydroxy quininib possessed anti-angiogenic properties to a much greater level than the parent compound quininib as evidenced by significantly reducing migration potential of human microvascular endothelial cells and tubule formation of human microvascular endothelial cells [22]. 1,4-dihydroxy quininib significantly decreased the expression of tyrosine-protein kinase receptor TIE-2 (TIE-2), and vascular cell adhesion protein (VCAM-1) in tumor conditioned media made from resected patient CRC tissue, and was well tolerated in mice [23]. Our drug development pipeline not only examines the anti-angiogenic properties of a compound, but also its simultaneous effects on the immune cell microenvironment. We are particularly interested in dendritic cells, whose antigen-presenting role is necessary to trigger a T cell response for tumor eradication. The maturation of DCs is essential for this process. Previously we showed patients who are poor responders to Bevacizumab treatment are deficient in inhibition of DC maturation markers, and this correlated with poor overall survival [24]. While other studies showed that VEGF can alter DC function thereby impacting on host immunity [25].

The goal of this study was to determine the potential beneficial effect of combining our novel anti-angiogenic small molecule (1,4-dihydroxy quininib) with current approved therapy such as Bevacizumab or 5-Fluoruoracil, Oxaliplatin and Leucovorin (FOLFOX) on a panel of angiogenic and inflammatory mediators and dendritic cell markers. We demonstrate that of a panel of 19 angiogenic and inflammatory serum markers tested, 1,4-dihydroxy quininib significantly altered the expression of the angiogenic marker TIE-2, angiopoietin 2 (a TIE-2 ligand), and inflammatory markers IL-6, IL-10 and IL-13 from resected human CRC ex vivo tissue explants. As the expression of VEGF family members (VEGF, VEGF-C, VEGF-D, PGF, FLT-1) was not significantly altered following treatment with 1,4-dihydroxy quininib, we hypothesize that 1,4-dihydroxy quininib is acting in an alternative pathway to the Bevacizumab targeting VEGF pathway, potentially via the angiopoietin-TIE-2 pathway. Additionally, we examine the effect of 1,4-dihydroxy quininib (both alone and in combination with Bevacizumab or FOLFOX) on the immune environment through its action on the maturation status of dendritic cells. We examined the expression levels of known phenotypic and maturation dendritic cell makers which included CD11c, CD86, CD83 and CD40 [26] and demonstrated a significant increase in CD11c and CD86 expression compared to control following treatment with 1,4-dihydroxy quininib alone and in combination with Bevacizumab following LPS maturation stimulus. Resistance to treatment [27], low rates of overall survival and progression free survival and toxicity profiles of current therapies [28] remain major challenges facing patients with mCRC. Combining 1,4-dihydroxy quininib with current therapy could offer an alternative treatment intervention, which requires further preclinical development.

## Methods

### TCM collection preparation and handling

Collection of colorectal tumor explants was previously described [23]. Ethical approval was granted by St. James’s Hospital and Adelaide, Meath and National Children’s Hospital Institutional Review Board and written informed consent was collected from all patients. Following the removal of a piece of colorectal tumor during the patient surgery, the tissue was stored in a tissue wash buffer (phosphate buffer saline (PBS) supplemented with 4 μg/ml fungizone (Gibco), 1% penicillin-streptomycin (Gibco) and 30 μg/ml gentamicin (Sigma). The explant pieces were thoroughly washed with additional tissue wash buffer within 30 min of the tissue being removed during surgery and stored in a 20% DMSO/HBSS buffer solution supplemented with 10% FBS, 2 μg/ml fungizone (Gibco) and 1% penicillin-streptomycin (Gibco) and stored at − 80 °C. Each piece of human CRC tumor explant was thawed at RT and placed in a petri dish containing wash buffer (PBS supplemented with 4 μg/ml fungizone (Gibco), 1% penicillin-streptomycin (Gibco) and 30 μg/ml gentamicin (Sigma). The tissue was washed thoroughly and cut into smaller pieces. Smaller pieces of tumor were washed three times for 5 min in fresh wash buffer before being placed into wells of a 24-well plate containing either (i) 0.1% DMSO, (ii) 1,4-dihydroxy quininib (10 μM), (iii) Bevacizumab (100 μg/ml) or (iv) FOLFOX (10 μM 5-Fluorouracil, 5 μM oxaliplatin and 2.5 μg/ml folinic acid), or a combination of (v) 1,4-dihydroxy quininib (10 μM) and Bevacizumab (100 μg/ml) or (vi) 1,4-dihydroxy quininib (10 μM) and FOLFOX (10 μM 5-Fluorouracil, 5 μM oxaliplatin and 2.5 μg/ml folinic acid) in warm RPMI-1640 medium supplemented with 10% FBS, 4 μg/ml fungizone (Gibco), 1% penicillin-streptomycin (Gibco) and 30 μg/ml gentamicin (Sigma). Explants were incubated for 72 h at 37 °C and 5% CO_2_. Plates were wrapped in parafilm to prevent evaporation of drug-medium during the incubation period. After 72 h, the tumor conditioned medium (TCM) was stored at − 20 °C and the residual explant tissue was snap-frozen in liquid nitrogen and stored at − 80 °C. The levels of secreted angiogenic and inflammatory factors from human CRC explants was determined by carrying out ELISAs on the tumor conditioned media collected following explant culture.

### Quantification of soluble factors by ELISA

Expression levels of Angiopoietin 1 and Angiopoietin 2 were measured in TCM from *n* = 7 patients (one pateint sample in each assay was below the level of detection) according to the manufacturer's instructions using the Human Angiopoietin 1/2 Quantikine ELISA kits from R&D systems. Multiplex ELISA kits (Meso-Scale Discovery) were used to according to the manufacturer’s instructions (neat TCM was used). The human Angiogenesis Panel 1 contained vascular endothelial growth factor (VEGF), VEGF-C, VEGF-D, tyrosine kinase (TIE)-2, vascular endothelial growth factor receptor (FLT)-1, placental growth factor (PGF) Gen B, and basic fibroblast growth factor (bFGF). The Pro-inflammatory Panel 1 contained interferon (IFN)-γ, interleukin (IL)-1β, IL-2, IL-4, IL-6, IL-8, IL-10, IL-12p70, IL-13 and tumor necrosis factor (TNF)-α. To normalize soluble factor amounts to the amount of starting tumor, patient tumor samples were harvested after TCM preparation, homogenised in ice-cold T-PER lysis reagent (Fisher) (proprietary detergent with 25 mM bicine and 150 mM sodium chloride (pH 7.6)) supplemented with 10 μl/ml protease inhibitor (Roche) and the BCA (Fisher) assay kit was used to quantify total protein extracted from explant tissue in μg/ml as per the manufacturer's instructions. Secretion levels of the angiogenic and inflammatory mediators were normalized to total protein content prior to statistical analysis.

### DC isolation and culture

Human monocyte-derived immature DCs were generated from peripheral blood mononuclear cells (PBMCs) obtained from buffy coat preparations (National Blood Centre, St. James’s Hospital, Dublin) by density gradient centrifugation (Lymphoprep) as described [29, 30]. Briefly, monocytes were isolated by positive selection using anti-CD14 magnetic microbeads as described by the manufacturer (Miltenyi Biotec) and seeded at a density of 1 × 10^6^ cells/ml in 6-well plates in 3 ml of RPMI-1640 medium containing 10% defined HyClone FBS (Thermo Scientific), 1% penicillin/streptomycin, 1% fungizone, human granulocyte macrophage colony-stimulating factor (50 ng/ml; Immunotools), and human IL-4 (70 ng/ml; Immunotools) in a humidified atmosphere with 5% CO_2_ at 37 °C. Cells were fed at day 3 by replacing half the medium made up with fresh cytokines. At day 6, CD11c^+^ cells exhibited an immature DC phenotype, as confirmed by ability to upregulate maturation markers following LPS stimulation.

### Stimulation of monocyte-derived DCs

Day 6 immature DCs were plated in 96-well plates at 2 × 10^5^ cells in 200 ml RPMI-1640 media supplemented with 10% defined, low-endotoxin Hyclone FBS (Thermo Scientific) and stimulated with a 1:2 dilution of conditioned media, or matched background media controls, for 4–5 h before exposure to 10 mg/ml of ultrapure TLR4 agonist *Escherichia coli* lipopolysaccharide (LPS-EB; Invivogen) overnight. Supernatants were harvested and frozen, and cells were assessed for expression of DC surface markers which included HLA-DR, CD11c, CD86, PDL1, CD40, CD80, CD83, and CD54.

### Flow cytometry

DCs were stained with the following antibodies: phycoerythrin (PE)- anti-CD80, PerCP-Cy5.5- anti-CD86, Pe-Cy7- anti-CD83, Brilliant Violet 421- anti-PD-L1, Brilliant Violet 510- anti-CD11c, allophycocyanin (APC)- anti-CD54, and APC-Cy7- anti-HLA-DR (Biolegend). Samples were acquired on DAKO CyAn flow cytometer (Beckman Coulter) with compensation performed with positive and negative compensation beads (BD Biosciences). The mean fluorescence intensity of markers expressed by CD11c^+^ cells were analyzed with FlowJo software (Tree Star Inc.).

### Statistical analysis

Statistical analysis was carried out using paired non-parametric Wilcoxon signed rank test in GraphPad Prism (version 8) to determine significance between groups. The R project for statistical computing (version 3.6.2) [31] and corrplot package was used to generate visualizations of correlations [32].

## Results

### Treatment of ex vivo human colorectal tumors with 1,4-dihydroxy quininib alone and in combination with Bevacizumab or FOLFOX reduces secretions of the angiogenic mediator TIE-2

**Fig. 1:**
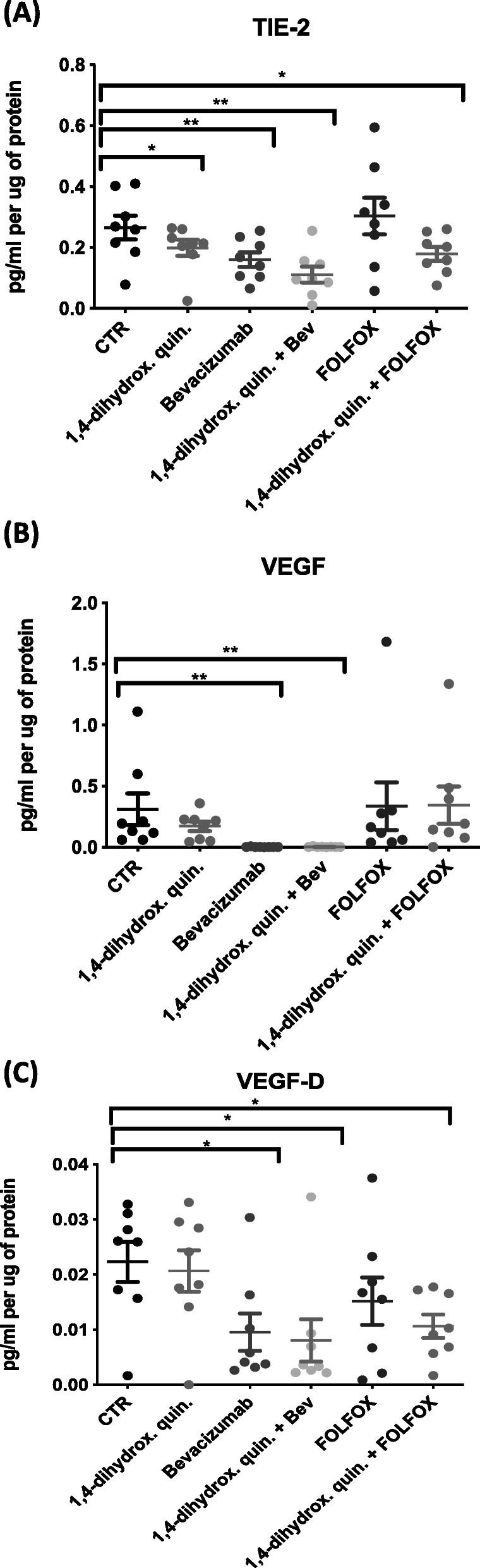
Expression of angiogenic mediators in tumor conditioned media from resected CRC tissue. The MSD V-plex Angiogenesis panel 1 was used to determine the expression level of 7 angiogenic markers in tumor conditioned media generated from resected patient CRC tissue (*n* = 8). **a** Treatment with 1,4-dihydroxy quininib or Bevacizumab alone was found to significantly decrease TIE-2 expression level (*p* < 0.05, FC = 1.2 and *p* < 0.01, FC = 1.7, respectively) and in combination with Bevacizumab (*p* < 0.01, FC = 2.7) or FOLFOX (*p* < 0.05, FC = 1.5) compared to control. **b** A significant reduction in VEGF expression was observed following treatment with Bevacizumab (*p* < 0.01, FC = 76.0) and 1,4-dihydroxy quininib combined with Bevacizumab (*p* < 0.01, FC = 58.2). **c** A significant reduction in VEGF-D expression was observed following treatment with Bevacizumab (*p* < 0.05, FC = 5.2), 1,4-dihydroxy quininib combined with Bevacizumab (*p* < 0.05, FC = 7.5) or FOLFOX (*p* < 0.05, FC = 2.7). Statistical analysis was performed using Wilcoxon signed rank test to determine significance between groups. (*p < 0.05, **p < 0.01). 1,4-dihydrox. Quin. = 1,4-dihydroxy quininib, Bev = Bevacizumab, FC = Fold Change. Error bars represent mean SEM. Non-significant results are found in Supplementary Figure 1

Following patient consent, surgically resected CRC tissues from eight patients (4 male, 4 female) were used for this study. The median age at the time of surgery was 74.5 years (range: 62–82 years). Additional characteristics of the patient cohort can be found in Supplementary Table 1. The expression levels of secreted angiogenic mediators from tumor explants into the tumor conditioned media (TCM) (treated with 1,4-dihydroxy quininib, Bevacizumab, FOLFOX, or a combination of 1,4-dihydroxy quininib with Bevacizumab or FOLFOX) was assessed by multiplex ELISA. Seven angiogenic markers (VEGF, VEGF-C, VEGF-D, TIE-2, FLT-1, PGF Gen B, and bFGF) were analyzed. The expression level of TIE-2 was significantly decreased following treatment with 1,4-dihydroxy quininib (*p* = 0.039, fold change (FC) = 1.2), Bevacizumab (*p* = 0.0078, FC = 1.7), 1,4-dihydroxy quininib in combination with Bevacizumab (*p* = 0.0078, FC = 2.7), and FOLFOX (*p* = 0.0391, FC = 1.5) versus control, Fig. 1a, Supplemental Table 2. Explants treated with 1,4-dihydroxy quininib alone did not significantly alter VEGF expression versus control, in comparison to Bevacizumab treatment (*p* = 0.0078, FC = 76.0), or, 1,4-dihydroxy quininib in combination with Bevacizumab (*p* = 0.0078, FC = 58.2) treatment which both significantly decreased expression levels versus control (Fig. 1b, Supplementary Table 2). Similarly, 1,4-dihydroxy quininib alone did not significantly alter VEGF-D expression versus control, compared to Bevacizumab alone (*p* = 0.0391, FC = 5.2), 1,4-dihydroxy quininib in combination with Bevacizumab (*p* = 0.0391, FC = 7.5), or, 1,4-dihydroxy quininib in combination with FOLFOX (*p* = 0.0234, FC = 2.7) treatment which significantly decreased expression levels versus control (Fig. 1c). No statistical significance versus control was observed for bFGF, PGF, FLT-1 and VEGF-C expression levels following drug treatments (Supplementary Fig. 1). These results suggest 1,4-dihydroxy quininib is acting in an alternate pathway to the Bevacizumab targeting VEGF pathway and decreases TIE-2 expression when combined with approved therapies Bevacizumab or FOLFOX.

### 1,4-dihydroxy quininib increases Angiopoietin 2 secretion levels in tumor conditioned media

**Fig. 2:**
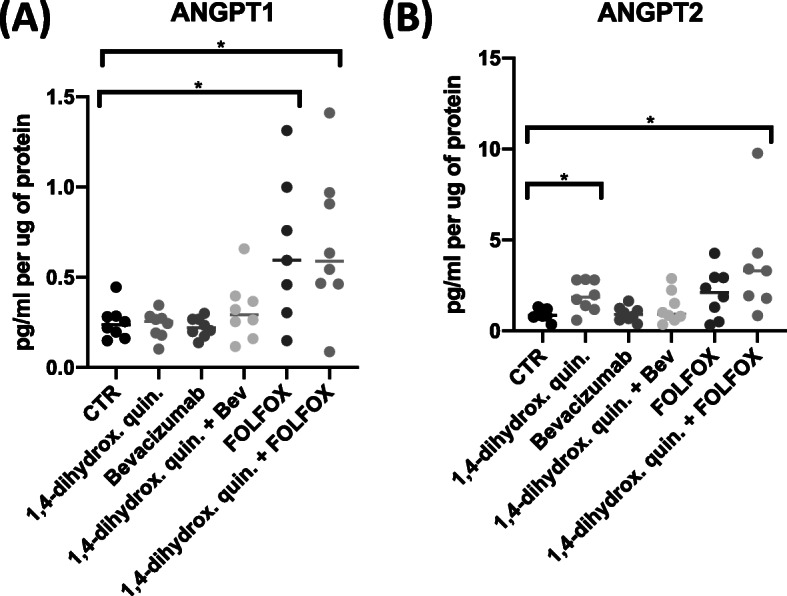
Expression of angiopoietin 1 & 2 in TCM from resected CRC tissue. Human Angiopoietin 1 and Human Angiopoietin 2 Quantikine ELISA kits from R&D systems were used to determine the expression of Angiopoietin 1 and Angiopoietin 2 in tumor conditioned media generated from resected patient CRC tissue. **a** The expression level of ANGPT1 significantly increased following treatment with FOLFOX (*p* < 0.05, FC = 2.5, *n* = 8) and 1,4-dihydroxy quininib in combination with FOLFOX (*p* < 0.05, FC = 2.5, *n* = 7) compared to control. **b** The expression level of ANGPT2 significantly increased following 1,4-dihydroxy quininib treatment (*p* < 0.05, FC = 2.2, *n* = 8), and 1,4-dihydroxy quininib treatment in combination with FOLFOX (*p* < 0.05, FC = 3.9, *n* = 7) compared to control. Statistical analysis was performed using a paired non-parametric Wilcoxon signed rank test to determine significance between groups. (*p < 0.05). 1,4-dihydrox. Quin. = 1,4-dihydroxy quininib, Bev = Bevacizumab, FC = Fold Change. Error bars represent mean SEM

To determine if 1,4-dihydroxy quininib is acting upstream of TIE-2 the secreted levels of angiopoietin ligands, angiopoietin 1 (ANGPT1) and angiopoietin 2 (ANGPT2) which act as agonists and antagonists of TIE-2 were analyzed. Expression levels of ANGPT1 and ANGPT2, were measured in tumor TCM following treatment with 1,4-dihydroxy quininib alone or in combination with Bevacizumab or FOLFOX. Treatment with 1,4-dihydroxy quininib alone did not significantly alter the expression of ANGPT-1 compared to control (Fig. 2a), however the expression level of ANGPT2 was significantly increased (*p* = 0.0234, FC = 2.2) compared to control (Fig. 2b). The expression level of ANGPT1 and ANGPT2 both significantly increased by 2.5-fold and 3.9-fold respectively, (*p* = 0.0156), in the 1,4-dihydroxy quininib + FOLFOX treatment groups compared to control.

The increased expression levels observed of ANGPT2 (a known TIE-2 antagonist) following 1,4-dihydroxy quininib treatment and the subsequent reduction of TIE-2 expression further supported our hypothesis that 1,4-dihydroxy quininib may be acting on the TIE-2 signaling pathway.

### 1,4-dihydroxy quininib significantly alters the expression of pro-inflammatory mediators in tumor conditioned media

**Fig. 3:**
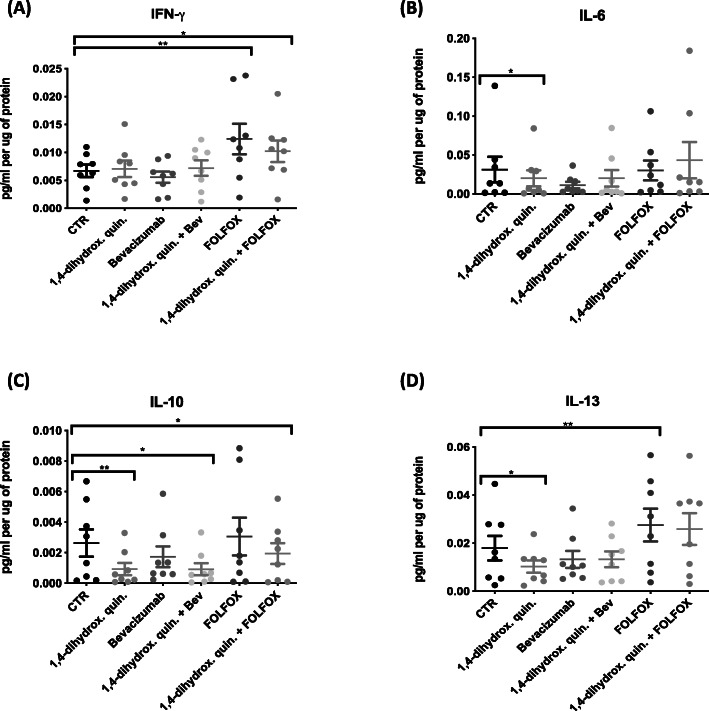
Expression of pro-inflammatory mediators in TCM from resected CRC tissue. The MSD V-plex Proinflammatory panel 1 was used to determine the expression level of 10 proinflammatory markers in tumor conditioned media generated from resected patient CRC tissue. **a** Treatment with FOLFOX and 1,4-dihydroxy quininib in combination with FOLFOX significantly increased IFN-γ expression (*p* < 0.01, FC = 1.6, and *p* < 0.05, FC = 1.5 respectively). **b** Treatment with 1,4-dihydroxy quininib significantly decreased the expression level of IL-6 (*p* < 0.05, FC = 2.3). **c** The expression level of IL-10 significantly decreased following treatment with 1,4-dihydroxy quininib (*p* < 0.01, FC = 5.1), 1,4-dihydroxy quininib in combination with Bevacizumab (*p* < 0.05, FC = 3.9), and 1,4-dihydroxy quininib in combination with FOLFOX (*p* < 0.05, FC = 1.3) compared to control. **d** The expression level of IL-13 significantly decreased following treatment with 1,4-dihydroxy quininib (*p* < 0.05, FC = 1.5) and significantly increased following treatment with FOLFOX (*p* < 0.01, FC = 1.8) compared to control. Statistical analysis was performed using a paired non-parametric Wilcoxon signed rank test to determine significance between groups. (*p < 0.05, **p < 0.01). 1,4-dihydrox. Quin. = 1,4-dihydroxy quininib, Bev = Bevacizumab, FC = Fold Change. Error bars represent mean SEM. Non-significant results are found in Supplementary Figure 2

The Pro-inflammatory multiplex ELISA panel containing 10 cytokines IFN-γ, IL-1β, IL-2, IL-4, IL-6, IL-8, IL-10, IL-12p70, IL-13 and TNF-α were quantified by multiplex ELISA (Fig. 3). Expression levels of IFN-γ significantly increased (*p* = 0.0078, FC = 1.6) following treatment with FOLFOX, and FOLFOX in combination with 1,4-dihydroxy quininib (*p* = 0.0391, FC = 1.5) compared to control (Fig. 3a). Treatment with 1,4-dihydroxy quininib alone had no effect on IFN-γ expression. Treatment with 10 µM 1,4-dihydroxy quininib caused a significant reduction in IL-6 (*p* = 0.0156, FC = 2.3, Fig. 3b), IL-10 (*p* = 0.0078, FC = 5.1, Fig. 3c) and IL-13 (*p* = 0.0391, FC = 1.5, Fig. 3d), expression compared to control. Combining 1,4-dihydroxy quininib with either Bevacizumab (*p* = 0.0391, FC = 3.9) or FOLFOX (*p* = 0.0156, FC = 1.3) treatment further significantly reduced the expression of IL-10 versus control compared to either treatment alone in our patient cohort (Fig. 3c). Supplementary Table 2 contains a summary of significant results. Following drug treatment, no significant difference in expression of IL-1β, IL-2, IL-4, IL-8, IL-12p70, and TNF-α was observed versus control (Supplementary Figure 2).

In summary, TCM treated with 10 µM 1,4-dihydroxy quininib significantly reduced the expression levels of IL-6, IL-10 and IL-13 compared to control. Neither Bevacizumab or FOLFOX alone significantly reduced IL-10 expression however when used in combination treatment with 1,4-dihydroxy quininib, the expression levels of IL-10 were significantly reduced compared to control.

As 1,4-dihydroxy quininib was found to significantly alter secreted expression levels of angiogenic and inflammatory mediators from tumor conditioned media we next wanted to determine the effect of 1,4-dihydroxy quininib on the immune environment.

### Treatment with 1,4-dihydroxy quininib significantly increases the expression of markers of dendritic cell maturation

**Fig. 4:**
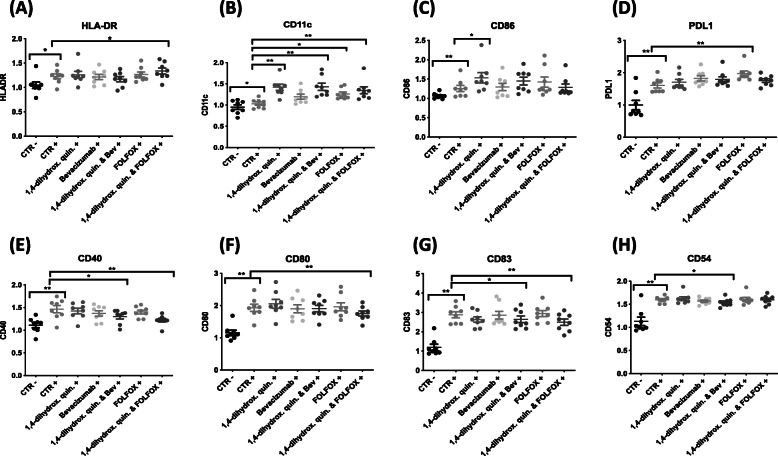
Affect of 1,4-dihydroxy quininib alone and in combination on dendritic cell markers. Monocyte-derived dendritic cells were co-cultured with tumor conditioned media generated from resected patient CRC tissue. Flow cytometry analysis was used to determine expression levels of 8 dendritic cell markers. Stimulation with LPS (to induce DC maturation) significantly increased the expression of all 8 DC surface markers (*p < 0.05, **p < 0.01). **a** Combination treatment of 1,4-dihydroxy quininib with FOLFOX significantly increased HLA-DR expression (*p* < 0.05, FC = 1.1), **b** The expression level of CD11c significantly increased compared to LPS control following treatment with 1,4-dihydroxy quininib (*p* < 0.01, FC = 1.4), 1,4-dihydroxy quininib combined with Bevacizumab (*p* < 0.01, FC = 1.1), FOLFOX (*p* < 0.05, FC = 1.2), and 1,4-dihydroxy quininib in combination with FOLFOX (*p* < 0.01, FC = 1.3). **c** CD86 expression significantly increased compared to LPS control (*p* < 0.05, FC = 1.2). **d** PDL-1 expression level significantly increased compared to LPS control following treatment with FOLFOX (*p* < 0.01, FC = 1.2). **e** The expression of CD40 significantly decreased compared to LPS control following treatment with 1,4-dihydroxy quininib in combination with Bevacizumab (*p* < 0.05, FC = 1.1) and 1,4-dihydroxy quininib in combination with FOLFOX (*p* < 0.01, FC = 1.2). **f** CD80 expression significantly decreased compared to LPS control following treatment with 1,4-dihydroxy quininib in combination with FOLFOX (*p* < 0.01, FC = 1.1). **g** CD83 expression significantly decreased compared to LPS control following treatment with 1,4-dihydroxy quininib in combination with Bevacizumab (*p* < 0.05, FC = 1.2), and 1,4-dihydroxy quininib in combination with FOLFOX (*p* < 0.01, FC = 1.1). **h** CD54 expression significantly decreased compared to LPS control following treatment with 1,4-dihydroxy quininib in combination with Bevacizumab (*p* < 0.05, FC = 1.1). Statistical analysis was performed using Wilcoxon signed rank test to determine significance between groups. (*p < 0.05, **p < 0.01). ‘-’ denotes no LPS stimulation while ‘+’ denotes LPS stimulation. 1,4-dihydrox. Quin. = 1,4-dihydroxy quininib, Bev = Bevacizumab, FC = Fold Change. Error bars represent mean SEM

To determine if proteins secreted from the different treated tumor microenvironments cross-talk with the ability of dendritic cells (DCs) to mature, tumor conditioned media from CRC explants were co-cultured with isolated immature DCs and a panel of known DC phenotypic and maturation markers were assessed. This panel included HLA-DR, PDL1, CD11c, CD40, CD54, CD80, CD83, and CD86. DCs were analyzed in the presence and absence of LPS, a known activator of DC maturation (Supplementary Figure 3 represents percentage positive cells of CD11c expression from 1 patient). Graphs represent control DC samples +/− LPS stimulation and LPS activated DC that were cultured in the presence of TCM that had been treated individually with 1,4-dihydroxy quininib, Bevacizumab, FOLFOX or a combination of 1,4-dihydroxy quininib with Bevacizumab or FOLFOX as previously described. Analysis of the DC control samples revealed LPS stimulation significantly increased the expression level of all 8 DC markers (HLA-DR 1.2-fold, CD11c 1.1-fold, CD86 1.2-fold, CD40 1.3-fold, CD80 1.7-fold, CD83 2.8-fold, PDL-1 1.9-fold, and CD54 1.6-fold). In the presence of LPS, TCM treated with 1,4-dihydroxy quininib significantly increased the known phenotypic DC marker CD11c (*p* = 0.0078, FC = 1.4), and the DC maturation marker CD86 (*p* = 0.0156, FC = 1.2), compared to control Fig. 4b&c. 1,4-dihydroxy quininib treatment alone had no effect on expression levels of HLA-DR, PDL-1, CD40, CD80, CD83, and CD54 compared to control (Fig. 4 a, d-h). 1,4-dihydroxy quininib in combination with Bevacizumab significantly increased the expression of CD11c (*p* = 0.0078, FC = 1.1) (Fig. 4b), while it significantly reduced the expression of CD40 (*p* = 0.0156, FC = 1.1, Fig. 4e), CD83 (*p* = 0.0234, FC = 1.2, Fig. 4g), and CD54 (*p* = 0.0469, FC = 1.1, Fig. 4h), compared to control. 1,4-dihydroxy quininib in combination with FOLFOX significantly increased HLA-DR (*p* = 0.03491, FC = 1.1, Fig. 4a) and CD11c (*p* = 0.0078, FC = 1.3, Fig. 4b) expression compared to control, while the expression levels of CD40, CD80, and CD83 all significantly decreased (all *p* = 0.0078, FC = 1.2, 1.1, and 1.1 respectively, Fig. 4e-g, Supplementary Table 2).

In summary, 1,4-dihydroxy quininib treatment alone significantly increases the expression of CD11c and CD86 compared to control while combination treatment of 1,4-dihydroxy quininib with Bevacizumab or FOLFOX significantly alters the expression levels of DC maturation markers.

### Expression levels of CD11c and CD86 correlate with inflammatory and angiogenic expression

**Fig. 5:**
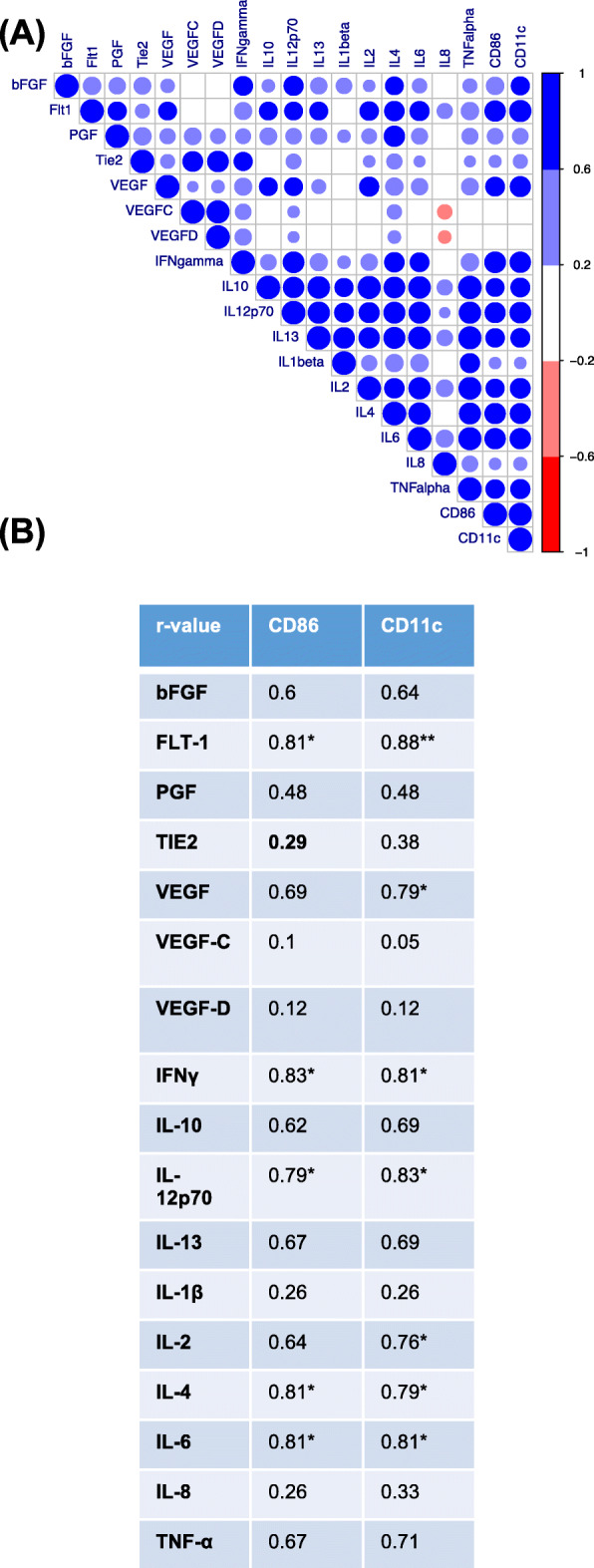
Dendritic cell markers CD86 and CD11c correlate with angigoenic and inflammatory mediators. Expression levels of angiogenic and inflammatory mediators from TCM was correlated with the expression levels of dendritic cell markers CD86 and CD11c. **a** Visualization of correlations using the ‘corrplot’ package for R (dark blue circles indicate spearman correlation values > 0.6). **b** A number of significant correlations existed such as CD86 and CD11c with FLT-1, IFN-γ, IL-12p70, IL-4 and IL-6 (*p* < 0.05). Correlation analysis conducted using GraphPad Prism software, significance of correlations indicated by *(*p* < 0.05) or**(*p* < 0.01)

As 1,4-dihydroxy quininib significantly increased the expression level of the phenotypic DC marker CD11c and maturation marker CD86 we correlated these results with 1,4-dihydroxy quininib treated patient TCM in which the raw expression levels of 7 secreted angiogenic and 10 inflammatory markers were calculated (Fig. 5). Strong correlations (*r* > 0.6) were observed for CD86 and CD11c with the inflammatory panel IFN-γ, IL-10, IL-12p70, IL-13, IL-2, IL-4, IL-6 and TNF-α. CD11c and CD86 both correlated (*r* > 0.6) with the angiogenic markers FLT-1 and VEGF-C. CD11c also correlated (*r* > 0.6) with bFGF. While strong correlations existed, significance (*p* < 0.05) was only detected between CD86 with FLT-1, IFN-γ, IL-4 and IL-6. CD11c significant interactions included FLT-1 (*p* < 0.01), VEGF, IFN-γ, IL-12p70, IL-2, IL-4, and IL-6 (all *p* < 0.05).

### Release of angiogenic and inflammatory mediators from dendritic cells

**Fig. 6:**
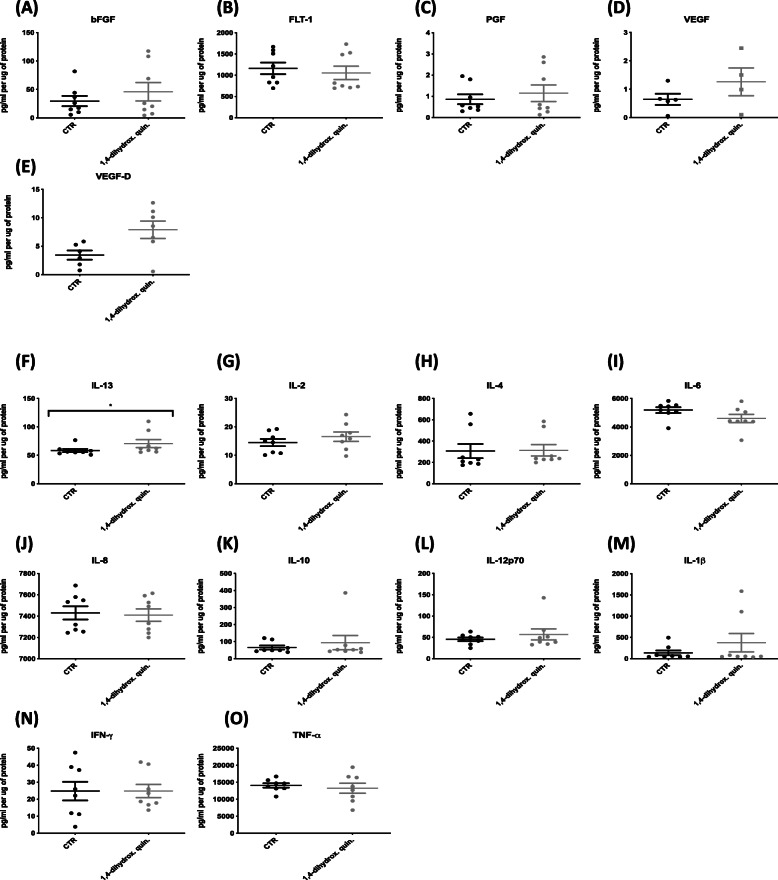
Release of angiogenic & inflammatory mediators from DC supernatant: Supernatant from co-culturing TCM and isolated monocyte-derived DCs was collected to determine **(a-e)** the expression of 7 angiogenic markers using the MSD V-plex angiogenesis and **(f-o)** expression of 10 inflammatory markers using the MSD V-plex inflammatory panel. The expression level of VEGF-C and TIE-2 were below the level of detection. **f** Treatment of 1,4-dihydroxy quininib (10 μM) significantly increased (*p* < 0.05, FC = 1.1) the expression level of IL-13 compared to vehicle control (0.1% DMSO). Statistical analysis was performed using Wilcoxon signed rank test to determine significance between groups, * = *p* < 0.05. 1,4-dihydrox. Quin. = 1,4-dihydroxy quininib, Bev = Bevacizumab, FC = Fold Change. Error bars represent mean SEM

To determine the secreted expression levels of angiogenic and inflammatory mediators from monocyte-derived DC supernatant from the LPS induced co-cultured DC-TCM was collected. As mentioned previously, the angiogenic panel consisted of VEGF, VEGF-C, VEGF-D, TIE-2, FLT-1, PGF Gen B, and bFGF while the inflammatory panel consisted of IFN-γ, IL-1β, IL-2, IL-4, IL-6, IL-8, IL-10, IL-12p70, IL-13 and TNF-α. The expression level of IL-13 was significantly increased following treatment with 10 µM 1,4-dihydroxy quininib compared to control (*p* = 0.0156, FC = 1.1) in the proinflammatory panel. No significant difference in expression was observed in the angiogenesis panel of bFGF, FLT-1, PGF, VEGF and VEGF-D with the expression levels of TIE-2 and VEGF-C below the level of detection (Fig. 6).

## Discussion

This study advanced our preclinical knowledge of our novel small molecule CysLT_1_ antagonist, 1,4-dihydroxy quininib in CRC. Cysteinyl leukotrienes are bioactive lipids derived from arachidonic acid [33] and through their receptors, CysLTs enhance vascular permeability, promote inflammation and angiogenesis [33, 34]. Target profiling demonstrated that similar to the parent compound quininib (Q1), 1,4-dihydroxy quininib is a CysLT_1_ antagonist [21, 22]. In colorectal adenocarcinoma, patients with high nuclear expression of CysLT_1_ have a worse outcome than patients with high nuclear CysLT_2_ expression [35]. This is demonstrated also in other cancers [36] highlighting a possible therapeutic benefit of targeting the cysteinyl leukotriene pathway via antagonists such as our novel small molecule 1,4-dihydroxy quininib.

We demonstrate that 1,4-dihydroxy quininib can significantly alter the expression of angiogenic and inflammatory mediators ex vivo from human ex vivo CRC tumor explant tissue. Of particular interest was the altered expression levels following combination treatment of 1,4-dihydroxy quininib with Bevacizumab or FOLFOX. For instance, a significant decrease in IL-10, a key cytokine released by tumor cells to suppress the host immune function by inhibition of dendritic cell differentiation and maturation in vitro [37], was observed following combination treatment of 1,4-dihydroxy quininib with Bevacizumab. While it is known that IL-4 and IL-13 share common functionality and equally a common receptor [38], we observed that 1,4-dihydroxy quininib significantly reduced the expression of IL-13 but not IL-4 in TCM. Increased expression levels of IL-13 have previously been observed in breast, oral squamous cell carcinoma and colorectal cancer [39]. IL-13 has been shown to activate tumor associated macrophages which in turn promote tumor cell metastasis and survival, indicating its potential as a therapeutic target [39]. Interestingly, analysis of IL-13 expression secreted by isolated DCs following incubation with TCM significantly increased following exposure to 1,4-dihydroxy quininib, in contrast to what was observed in the TCM only experiment. DCs have previously been shown to produce IL-13 and its exposure is crucial in producing a T helper (Th2) cytokine response following exposure to allergens [40]. However, the effect of over-expression of IL-13 may alter in different tumor types and in some instances its secretion can enhance tumor growth [41].

Angiopoietin 1 & 2 (ANGPT1, ANGPT2) are the best characterized ligands of the receptor tyrosine kinase TIE-2. ANGPT1 functions as a TIE-2 agonist while ANGPT2 has dual functionality both as a TIE-2 antagonist and agonist, its function is context dependent [42, 43]. In the presence of the ANGPT2 regulator vascular endothelial protein tyrosine phosphatase (VE-PTP), ANGPT2 functions as an antagonist in blood endothelial cells, while in the absence of VE-PTP it functions as an agonist in lymphatic endothelial cells. The precise mechanism of how VE-PTP regulates ANGPT2 remains unclear [44, 45]. TIE-2 is known to be expressed by endothelial cells and at a lower level by pericytes [46]. TIE-2 expressing monocytes (TEMs) have also been observed in the blood and tumor and demonstrate a critical role for TEMS in angiogenesis and tumor progression [47]. Combining our novel anti-angiogenic small molecule 1,4-dihydroxy quininib with either Bevacizumab or FOLFOX also displayed a significant reduction in TIE-2 expression levels indicative of complementary inhibition. Targeting multiple pathways through the combination of 1,4-dihydroxy quininib and approved therapy may lead to improved overall outcome in the CRC patient population. Combination treatment of drugs targeting the Angiopoietin-TIE-2 pathway with anti-angiogenic VEGF-A therapy in an oncological and ophthalmological setting are currently in clinical development and combination treatment has been shown to outweigh single pathway targeting [19, 48]. Additionally, there is evidence which demonstrates a relationship between TIE-2 expression levels, and Bevacizumab treatment. Analysis of pre-chemotherapy plasma ANGPT1 and TIE-2 expression from patients participating in an ovarian cancer trial, randomized to conventional dose Carboplatin and Paclitaxel ± Bevacizumab is described by Backen et al., [49]**.** Their study revealed that a subgroup of patients in the experimental study arm who were receiving Bevacizumab treatment and who had high ANGPT1 and low TIE-2 expression had significantly improved progression free survival compared to conventional treatment. Analysis of a panel of circulating and imaging biomarkers from patients with metastatic colorectal during treatment with Bevacizumab followed by cytotoxic chemotherapy and Bevacizumab demonstrated that TIE-2 is a tumor vascular response biomarker for Bevacizumab in patients with metastatic colorectal cancer. Furthermore, the study suggested TIE-2 expression should be monitored clinically to optimize VEGF inhibitor use [50].

We have demonstrated that 1,4-dihydroxy quininib significantly increased the expression of ANGPT2 and reduced the expression of TIE-2. The function of ANGPT2 is context-dependent, with increased ANGPT2 levels found in diseases associated with vascular leak and inflammation [45]. The limitations of this study is in the small sample size and further investigation on the significant increase in ANGPT2 expression following 1,4-dihydroxy quininib treatment is warranted in larger cohorts. However, a reduction in TIE-2 expression was also observed when the sample size was increased to *n* = 15 in a single drug-study analysis of 1,4-dihydroxy quininib, as previously reported [23]. Furthermore, in this larger patient cohort we also showed a significant reduction in the expression of the surface adhesion molecule VCAM-1 and hypothesized that 1,4-dihydroxy quininib is acting on the angiopoietin-TIE signalling pathway [23]. Binding of VCAM-1 to the leukocyte integrin VLA-4 (very-late antigen-4) on leukocytes initiates their migration to regions of inflammation [51]. Kim et al., have previously demonstrated that ANGPT1 can suppress the VEGF-mediated induced expression of the adhesion molecules VCAM-1, ICAM-1 and E-selectin and reduce the VEGF induced adhesion of leukocytes to HUVECs [52].

Dendritic cell (DC) maturation is an essential process to ensure effective antigen presentation to T cells to activate an effector T cell response and eradicate tumors [53]. Tumor associated DCs can contribute to immune suppression in cancer if their functional activity is impaired [54]. A failure of DCs to mature, or inhibition of DC maturation by a drug can therefore have a detrimental effect to the patient. VEGF-A which is ubiquitous in the tumor microenvironment, can inhibit DC maturation via inhibition of NFκB [55]. While the anti-angiogenic drug Bevacizumab is well known to target the VEGF pathway, thereby decreasing the ability of VEGF-A to inhibit DC maturation, we have previously shown that patients who are resistant to this approved therapy display an impairment in dendritic cell maturation [24]. Using the known stimulant LPS to induce the maturation of dendritic cells in the presence of TCM, we have shown that treatment of patient explants with 1,4-dihydroxy quininib significantly increased the expression levels of the phenotypic DC marker CD11c and maturation marker CD86. The expression of CD11c, one of the key markers which differentiates dendritic cells from other leukocytes did not significantly alter following treatment with Bevacizumab, however, when Bevacizumab was combined with 1,4-dihydroxy quininib a significant increase in CD11c expression was observed, indicating a beneficial combined effect. A similar beneficial effect was observed in HLA-DR expression levels following 1,4-dihydroxy quininib and FOLFOX combination treatment. However, other known markers of DC maturation whose expression level was assessed in combination treatment (CD40, CD54, CD80 and CD83) showed significant decreasing expression levels. Previously, we also demonstrated a reduction in CD83 expression when monocyte derived DCs were cultured in Bevacizumab conditioned media (ex vivo CRC tumor tissue was cultured in media +/− Bevacizumab) and this inhibition of LPS-induced CD83, expression significantly correlated with poorer clinical outcome [56]. The exact reason why the expression levels of these markers in particular decreased following combination of 1,4-dihydroxy quininib with Bevacizumab or FOLFOX treatment is as of yet unknown. Possibly, it could be due to the simultaneous timing of drug administration to the explant tissue to generate TCM. Additional analysis in a larger patient cohort, potentially using stepwise administration of our drug combinations is required to fully understand the role of 1,4-dihydroxy quininib on DC maturation.

Correlation analysis of the two lead DC markers CD11c and CD86 with 1,4-dihydroxy quininib treated TCM angiogenic and proinflammatory panels revealed that strong significant correlations existed such as FLT-1 in the angiogenic panel, and numerous members of the proinflammatory panel, namely IFN-γ, IL-12p70, IL-4, and IL-6. Since DC maturation can be activated in response to inflammatory mediators it is unsurprising to see such strong correlations with CD86 and CD11c with the proinflammatory panel.

## Conclusions

In this study we have demonstrated that our novel anti-angiogenic small molecule 1,4-dihydroxy quininib significantly alters the expression of angiogenic and inflammatory mediators when combined with current approved therapies. Furthermore, combination treatment of 1,4-dihydroxy quininib with Bevacizumab or FOLFOX in the tumor microenvironment increases the expression levels of DC phenotypic and maturation markers. Both here, and in previous studies we hypothesized that 1,4-dihydroxy quininib is acting on the angiopoietin-TIE-2 pathway [23]**.** As we advance our preclinical knowledge of 1,4-dihydroxy quininib further in vivo combination studies are required to ascertain the full potential and mechanism of action 1,4-dihydroxy quininib as a combinational therapy in CRC.

## Supplementary information

**Additional file 1 Table S1.** Patient characteristics including surgical details, and Tumor-Nodal-Metastasis (TNM) stage. The median age at the time of surgery 74.5 years (range: 62–82 years), with an equal number of males [4] & females [4]

**Additional file 2 Table S2.** Summary of significant results including fold-change.

**Additional file 3 Figure S1.** Expression of angiogenic mediators in tumor conditioned media from resected CRC tissue. The MSD V-plex Angiogenesis panel 1 was used to determine the expression level of angiogenic markers in tumor conditioned media generated from resected patient CRC tissue (*n* = 8). Following drug treatment, no significant difference in expression level was observed for (A) bFGF, (B) PGF, (C) FLT-1, and (D) VEGF-C.

**Additional file 4 Figure S2.** Expression of pro-inflammatory mediators in TCM from resected CRC tissue. The MSD V-plex Proinflammatory panel 1 was used to determine the expression level of proinflammatory markers in tumor conditioned media generated from resected patient CRC tissue. Following drug treatment, no significant difference in expression level was observed for (A) IL-1β, IL-2, IL-4, IL-8, IL-12p70, and TNF-α.

**Additional file 5 Figure S3.** Flow Cytometry Analysis: **A** The experimental outline is illustrated describing the incubation of (i) DC preparations for the specified time and (ii) the conditions to which the cells were exposed, specifically IL-4 and GM-CSF cytokines to derive the DCs, conditioned media from the TCM to precondition the DCs and LPS to mature the DCs. Finally, DCs were analyzed by flow cytometry and DC supernatants were analyzed by ELISA. **B-C** The gating strategy of the monocyte-derived DCs is shown of singlet, monogate cells which are CD11c + (B) and the Fluorescence Minus One staining controls (C).

## Data Availability

Data and material presented in this article and in the supplementary information are available upon reasonable request from the corresponding author.

## Abbreviations

1,4-dihydrox. Quin.: 1,4-dihydroxy quininib
5-FU: Fluorouracil
ANGPT1: Angiopoietin-1
ANGPT2: Angiopoietin-2
BEV: Bevacizumab
bFGF: Basic Fibroblast Growth Factor
CRC: Colorectal Cancer
CysLT: Cysteinyl Leukotriene
DC: Dendritic Cell
DMSO: Dimethyl Sulfoxide
EGFR: Epidermal Growth Factor Receptor
ELISA: Enzyme Linked Immunosorbent Assay
FDA: Food and Drug Administration
FGF: Fibroblast Growth Factor
FLT-1: Vascular Endothelial Growth Factor Receptor-1
FOLFOX: Folinic acid, Fluorouracil, Oxaliplatin
HBSS: Hanks Balanced Salt Solution
HUVEC: Human Umbilical Vein Endothelial Cells
ICAM-1: Intracellular Adhesion Molecule-1
IFN-γ: Interferon gamma
IL: Interleukin
mCRC: Metastatic Colorectal Cancer
PBS: Phosphate Buffer Saline
PBMC: Peripheral Blood Mononuclear Cells
PE: Phycoerythrin
PGF: Placental Growth Factor
TEM: TIE2 Expressing Monocytes
TCM: Tumor Conditioned Media
Th2: T helper
TIE-2: Tyrosine Kinase-2
TNF-α: Tumor Necrosis Factor Alpha
VCAM-1: Vascular Cell Adhesion Molecule-1
VEGF: Vascular Endothelial Growth Factor
VE-PTP: Vascular Endothelial Protein Tyrosine Kinase
VLA-4: Very Late Antigen 4
