# Supplementary material for: Combining 1,4-dihydroxy quininib with Bevacizumab/FOLFOX alters angiogenic and inflammatory secretions in ex vivo colorectal tumors
Source: BMC Cancer. 2020 Oct 2;20:952. doi: 10.1186/s12885-020-07430-y (PMC7532092; doi:10.1186/s12885-020-07430-y)
Supplement: Supplementary file 1 — Additional file 1 Table S1. Patient characteristics including surgical details, and Tumor-Nodal-Metastasis (TNM) stage. The median age at the time of surgery 74.5 years (range: 62–82 years), with an equal number of males [4] & females [4] [file 12885_2020_7430_MOESM1_ESM.docx]

| Patient | TNM Stage | DUKES Stage | Surgery Type |
| --- | --- | --- | --- |
| A | T4N0 | B | Hemicolectomy |
| C | T4N1 | C1 | Right hemicolectomy |
| D | T4N0 | B | Right hemicolectomy |
| E | T1N0 | A | Proctectomy |
| F | T4N1 | C1 | Right hemicolectomy |
| G | T3N0 | B | Low anterior resection |
| H | T4N0 | B | Right hemicolectomy |
| I | T3N0 | B | Right hemicolectomy |

**SUPPLEMENTARY TABLE 1**
