# Supplementary material for: Combining 1,4-dihydroxy quininib with Bevacizumab/FOLFOX alters angiogenic and inflammatory secretions in ex vivo colorectal tumors
Source: BMC Cancer. 2020 Oct 2;20:952. doi: 10.1186/s12885-020-07430-y (PMC7532092; doi:10.1186/s12885-020-07430-y)
Supplement: Supplementary file 2 — Additional file 2 Table S2. Summary of significant results including fold-change. [file 12885_2020_7430_MOESM2_ESM.docx]

|  | **1,4-dihydroxy quininib** | | **Bevacizumab** | | **1,4-dihydroxy quininib + Bevacizumab** | | **FOLFOX** | | **1,4-dihydroxy quininib + FOLFOX** | |
| --- | --- | --- | --- | --- | --- | --- | --- | --- | --- | --- |
|  | **p** | **FC** | **p** | **FC** | **p** | **FC** | **p** | **FC** | **p** | **FC** |
|  | **FIGURE 1** | | | | | | | | | |
| **Fig.1 (A)**  **TIE-2** | **0.0391** | **1.2** | **0.0078** | **1.7** | **0.0078** | **2.7** | 0.5469 | 1.12 | **0.0391** | **1.5** |
| **Fig.1 (D)**  **VEGF** | 0.3125 | 1.2 | **0.0078** | **76.0** | **0.0078** | **58.2** | 0.9453 | 1.2 | 0.4609 | 1.0 |
| **Fig.1 (E)**  **VEGF-D** | 0.1953 | 1.2 | **0.0391** | **5.2** | **0.0391** | **7.5** | 0.1953 | 1.6 | **0.0234** | **2.7** |
|  | **FIGURE 2** | | | | | | | | | |
| **Fig.2 (A) ANGPT1** | ns | 1.0 | 0.0547 | 1.0 | 0.2500 | 1.2 | **0.0312** | **2.5** | **0.0156** | **2.5** |
| **Fig.2 (B) ANGPT2** | **0.0234** | **2.2** | 0.9453 | 1.0 | 0.3828 | 1.1 | 0.0547 | 2.5 | **0.0156** | **3.9** |
|  | **FIGURE 3** | | | | | | | | | |
| **Fig.3 (A)**  **IFN-g** | ns | 1.1 | 0.2500 | 1.2 | 0.9453 | 1.1 | **0.0078** | **1.6** | **0.0391** | **1.5** |
| **Fig.3 (E)**  **IL-6** | **0.0156** | **2.3** | 0.1094 | 2.5 | 0.1953 | 3.2 | 0.3828 | 1.3 | 0.1484 | 1.1 |
| **Fig.3 (F)**  **IL-13** | **0.0391** | **1.5** | 0.3828 | 1.4 | 0.1953 | 1.1 | **0.0078** | **1.8** | 0.1094 | 1.9 |
| **Fig.G (H)**  **IL-10** | **0.0078** | **5.1** | 0.3125 | 2.2 | **0.0391** | **3.9** | 0.8438 | 1.4 | **0.0156** | **1.3** |
|  | **FIGURE 4** | | | | | | | | | |
| **Fig.4 (A)**  **HLA-DR** | 0.8125 | 1.0 | 0.8438 | 1.0 | 0.0547 | 1.1 | 0.1484 | 1.0 | **0.0391** | **1.1** |
| **Fig.4 (B)**  **CD11c** | **0.0078** | **1.4** | 0.1094 | 1.1 | **0.0078** | **1.1** | **0.0156** | **1.2** | **0.0078** | **1.3** |
| **Fig.4 (C)**  **CD86** | **0.0156** | **1.2** | ns | 1.0 | 0.0781 | 1.2 | 0.4609 | 1.0 | 0.5469 | 1.1 |
| **Fig.4 (D)**  **PDL-1** | 0.2500 | 1.0 | 0.0781 | 1.1 | 0.0781 | 1.1 | **0.0078** | **1.2** | 0.3125 | 1.2 |
| **Fig.4 (E)**  **CD40** | 0.3828 | 1.0 | 0.1094 | 1.1 | **0.0156** | **1.1** | 0.2500 | 1.1 | **0.0078** | **1.2** |
| **Fig.4 (F)**  **CD80** | 0.2500 | 1.0 | 0.5469 | 1.1 | 0.7422 | 1.0 | 0.6719 | 1.0 | **0.0078** | **1.1** |
| **Fig.4 (G)**  **CD83** | 0.0781 | 1.1 | ns | 1.1 | **0.0234** | **1.2** | 0.2969 | 1 | **0.0078** | **1.1** |
| **Fig.4 (H)**  **CD54** | 0.7734 | 1.0 | 0.3125 | 1.0 | **0.0469** | **1.1** | ns | 1.0 | 0.9375 | 1.0 |

**Supplementary Table 2:** Summary of significant results compared to control. Significant results highlighted in bold
