# Supplementary figures and images for: Combining 1,4-dihydroxy quininib with Bevacizumab/FOLFOX alters angiogenic and inflammatory secretions in ex vivo colorectal tumors
Source: BMC Cancer. 2020 Oct 2;20:952. doi: 10.1186/s12885-020-07430-y (PMC7532092; doi:10.1186/s12885-020-07430-y)

SUPPLEMENTARY FIGURE 1

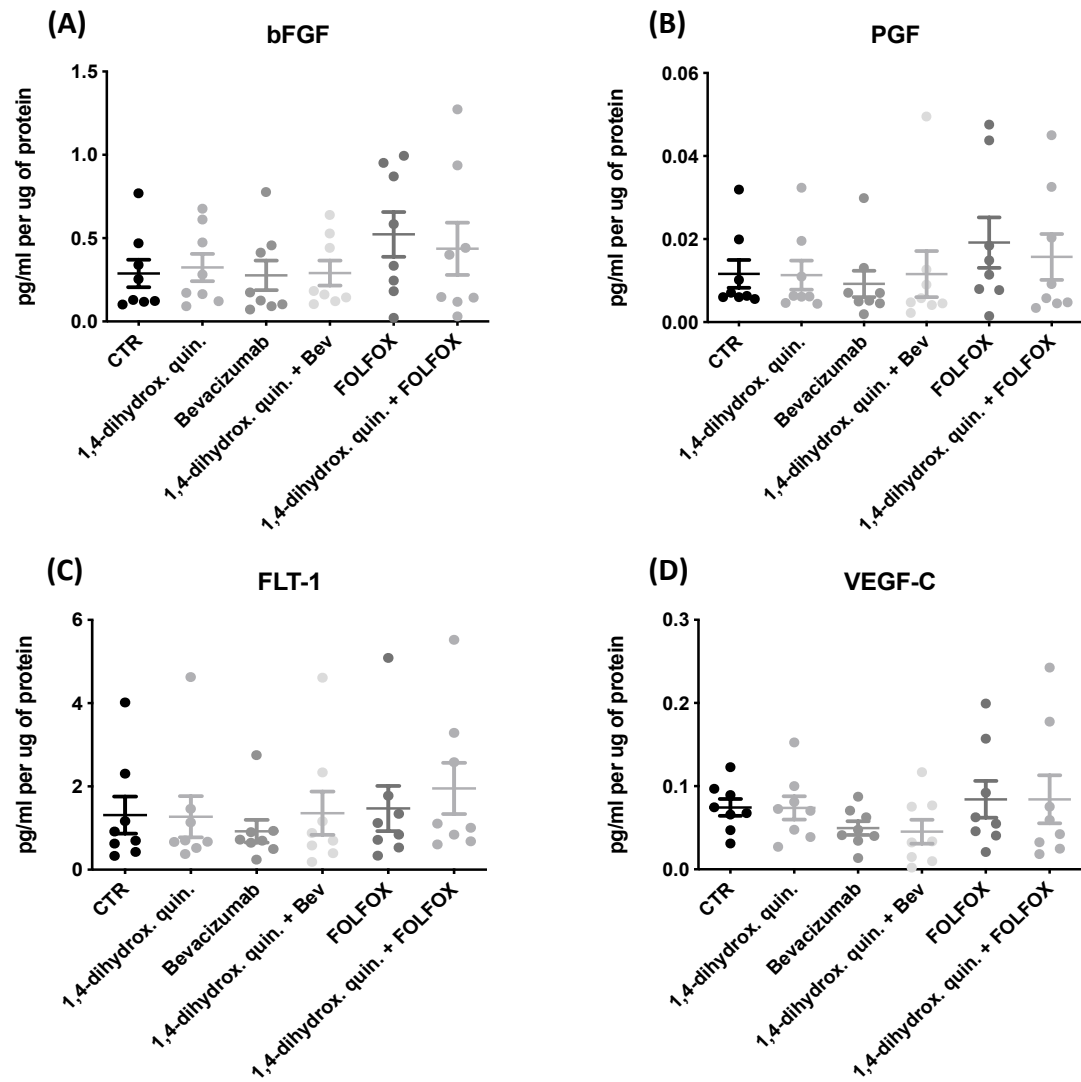

Supplement: Supplementary file 3 — Additional file 3 Figure S1. Expression of angiogenic mediators in tumor conditioned media from resected CRC tissue. The MSD V-plex Angiogenesis panel 1 was used to determine the expression level of angiogenic markers in tumor conditioned media generated from resected patient CRC tissue (n = 8). Following drug treatment, no significant difference in expression level was observed for (A) bFGF, (B) PGF, (C) FLT-1, and (D) VEGF-C. [file 12885_2020_7430_MOESM3_ESM.pdf]

## SUPPLEMENTARY FIGURE 2

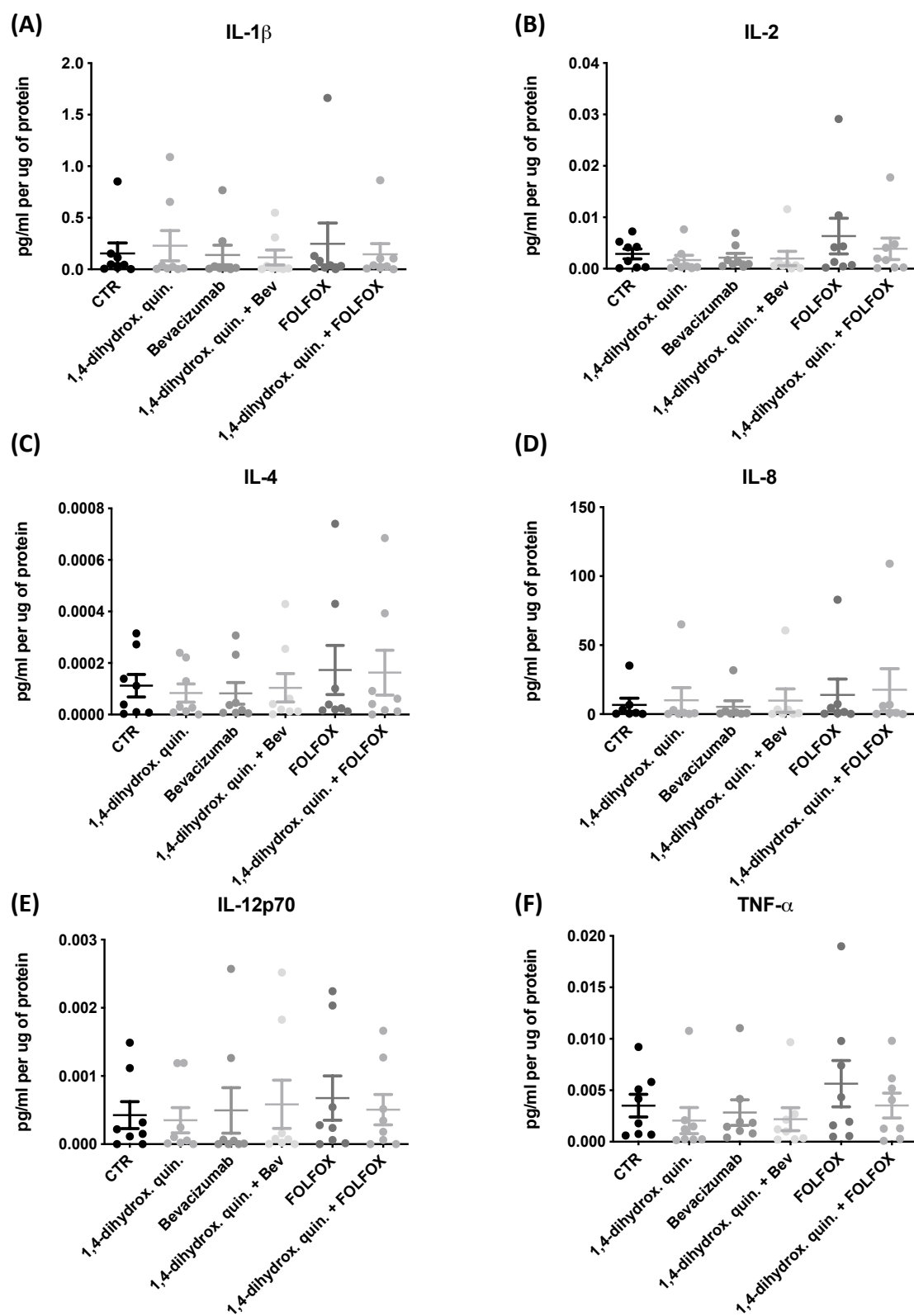

Supplement: Supplementary file 4 — Additional file 4 Figure S2. Expression of pro-inflammatory mediators in TCM from resected CRC tissue. The MSD V-plex Proinflammatory panel 1 was used to determine the expression level of proinflammatory markers in tumor conditioned media generated from resected patient CRC tissue. Following drug treatment, no significant difference in expression level was observed for (A) IL-1β, IL-2, IL-4, IL-8, IL-12p70, and TNF-α. [file 12885_2020_7430_MOESM4_ESM.pdf]

# SUPPLEMENTARY FIGURE 3

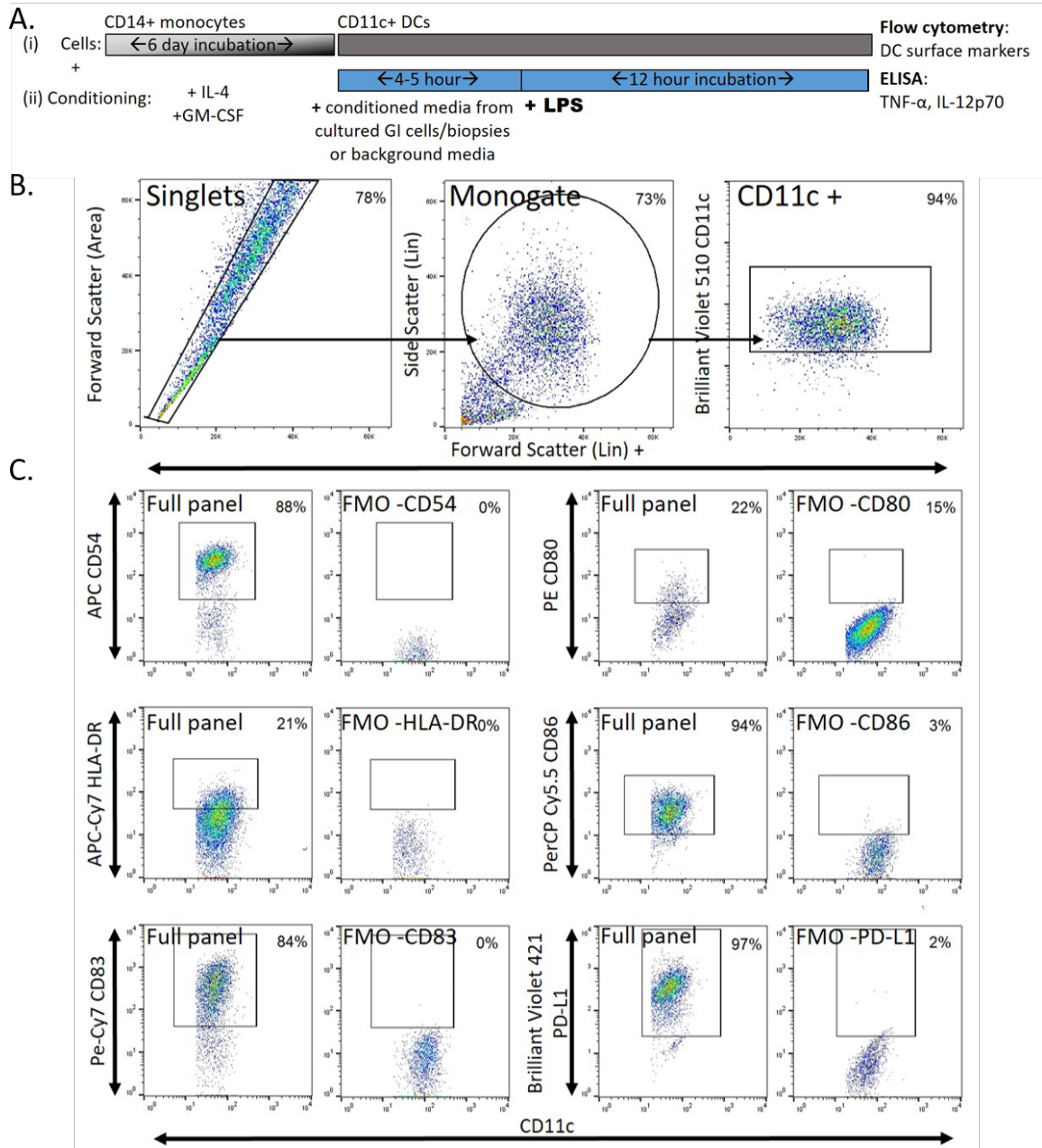

Supplement: Supplementary file 5 — Additional file 5 Figure S3. Flow Cytometry Analysis: A The experimental outline is illustrated describing the incubation of (i) DC preparations for the specified time and (ii) the conditions to which the cells were exposed, specifically IL-4 and GM-CSF cytokines to derive the DCs, conditioned media from the TCM to precondition the DCs and LPS to mature the DCs. Finally, DCs were analyzed by flow cytometry and DC supernatants were analyzed by ELISA. B-C The gating strategy of the monocyte-derived DCs is shown of singlet, monogate cells which are CD11c + (B) and the Fluorescence Minus One staining controls (C). [file 12885_2020_7430_MOESM5_ESM.pdf]
